# Royal Eye Hospital. Southwark

**Published:** 1892-12-10

**Authors:** 


### NEW HOSPITALS.

### ROYAL EYE HOSPITAL, SOUTHWARK.

The opening of the new premises of the Royal Eye Hospital, Southwark, by H.R.H. the Duke of York, is fixed for one p.m. on Thursday the 15th inst. This is likely to prove a memorable occasion in South London, which, though a district not less poverty stricken than the East-end, is one which until the foundation-stone of this hospital was laid by H.R.H. the Prince of Wales in 1890, had hardly any recollection of a Royal visit. The coming ceremonial, marking as it will the commencement of public life by the Duke of York in his present exalted position, and as the first Royal ceremonial of the year, excites peculiar enthusiasm. This will be intensified by a knowledge of the exceptionally special care bestowed upon all details in the design and fitting of this hospital. Sir Joseph Lister, Bart., has recently critically inspected the new premises throughout, and expressed his astonishment and gratification that London now possesses a hospital in which such conspicuous attention has been given to all the lessons revealed by the light which this parent and prophet of antiseptic surgery has shed for the benefit of suffering humanity, and the world-wide credit of British surgical research. His approval has taken lasting form, insomuch that Sir Joseph Lister has now cheerfully accepted, as an esteemed honour, the invitation of the authorities to become consulting surgeon to the institution, which, though a purely honorary office, is felt to be far from an undistinguished one in connection with a hospital which is the very embodiment of all that the Victorian era has taught regarding hospital construction.

The uniform and scrupulous practice of this hospital's authorities in not spending, other than for the relief of the suffering poor, any of the money entrusted to them, is now receiving tangible evidence of approval, for unsolicited, but most welcome, contributions are reaching the Chairman of the Rebuilding Sub-Committee, so that he hopes to have in hand sufficient to defray the expenses of the opening ceremonial without stinting in anything which can enhance the success, interest, and instructiveness of the occasion.

Arrangements are being made to comfortably seat a large company, who will assemble to the music of the Royal Marine Artillery Band. The special features of the hospital will be demonstrated by the aid of a powerful optical lantern, as without this device the many useful lessons which this hospital illustrates would be lost to the majority. The Reception Committee, which will be presided over by H.R.H. the Duke of Cambridge, as chairman, and Admiral of the Fleet, the Hon. Sir Harry Keppel, as vice-chairman, includes, among other notable and representative persons, the following: The Right Hon. the Lord Mayor, Lord Bishop of Rochester, Earl Kilmorey, Bishop Suffragan of Southwark, Archbishop of Westminster, Sir Thomas Lucas, Bart., Sir Joseph Fayrer, K.C.S.I., M.D., and Sir Richard Wyatt.

